# Impact of hyperhydration on fluid overload and hematopoietic cell transplant after post-transplant cyclophosphamide-based graft-versus-host-disease prophylaxis

**DOI:** 10.3389/fimmu.2025.1543099

**Published:** 2025-02-20

**Authors:** Diana T. Samuels, Janny M. Yao, Yazeed Samara, Dongyun Yang, Sally Mokhtari, Katrin Tiemann, Salman Otoukesh, Shukaib Arslan, Hoda Pourhassan, Stephanie Wu, Amanda Blackmon, Vaibhav Agrawal, Idoroenyi Amanam, Haris Ali, Amandeep Salhotra, Ibrahim Aldoss, Brian Ball, Paul Koller, Ahmed Aribi, Karamjeet Sandhu, Vinod Pullarkat, Andrew Artz, Eileen Smith, Forrest Stewart, Pamela Becker, Anthony Stein, Guido Marcucci, Stephen J. Forman, Ryotaro Nakamura, Monzr M. Al Malki

**Affiliations:** ^1^ Department of Pharmacy, City of Hope National Medical Center, Duarte, CA, United States; ^2^ Department of Hematology and Hematopoietic Cell Transplantation, City of Hope National Medical Center, Duarte, CA, United States; ^3^ Department of Computational and Quantitative Medicine, Division of Biostatistics, City of Hope National Medical Center, Duarte, CA, United States; ^4^ Department of Clinical and Translational Project Development, City of Hope National Medical Center, Duarte, CA, United States; ^5^ Department of Internal Medicine, Division of Cardiology, City of Hope National Medical Center, Duarte, CA, United States

**Keywords:** allogeneic hematopoietic cell transplantation, post-transplant cyclophosphamide, hyperhydration, hemorrhagic cystitis, fluid overload, fluid retention, fluid toxicity, weight gain

## Abstract

**Introduction:**

Hemorrhagic cystitis (HC) is an early complication after hematopoietic cell transplant (HCT) with post-transplant cyclophosphamide (PTCy). Hyperhydration can reduce HC, but may lead to fluid overload (FO), which has been associated with higher non-relapse mortality (NRM) after HCT.

**Methods:**

The objectives of this study were to grade FO between days 3 and 8 based on weight gain, diuretic therapy, and FO-related organ dysfunction and analyze the impact of FO on non-relapse mortality (NRM) and subsequently on overall survival (OS) of patients undergoing HCT with PTCy-based GvHD prophylaxis.

**Results:**

Two hundred seventy-five patients who received PTCy at City of Hope from 2009 to 2018 were included. A majority, 270 (98%) patients were diagnosed with early FO from day 3-8 post HCT, of whom 248 (92%) experienced mild to moderate (grade 1-2) FO, and 22 (8%) experienced severe (grade 3-4) FO. Day 100 NRM was significantly higher in patients with grade 3-4 FO compared to patients with grade 0-1 (59.1 vs 1.7%, CI: 0.006-0.053p<0.001) and grade 2 (59.1 vs 8.8%, CI: 0.043-0.178, p<0.001) FO. At 2 years, OS and DFS were significantly lower in patients who experienced grade 3-4 FO compared to patients who had grade 0-1 FO (31.8% vs 68.2%, CI: 0.616-0.755, p<0.001) and grade 2 FO (31.8% vs 62.5%; CI: 0.527-0.741, p<0.001). Additionally, each 5% weight gain from baseline was associated with higher NRM (HR=1.91, 95%CI: 1.64-2.23, p<0.001).

**Conclusion:**

Almost all patients undergoing hyperhydration for PTCy-induced HC will present with FO. Grade 3-4 FO is uncommon and associated with poor clinical outcomes. Weight gain could be used as an early and possibly modifiable indicator of FO.

## 1 Introduction

In the last decade, the use of post-transplant cyclophosphamide (PTCy) as graft-versus-host disease (GvHD) prophylaxis has increased. PTCy prophylaxis has been shown to effectively reduce the incidence of both severe acute GvHD (aGvHD) and chronic GvHD (cGvHD) in haploidentical hematopoietic cell transplant (HCT) and recently in the matched and mismatched unrelated donor setting (1–9).

Upon administration, cyclophosphamide is metabolized into nitrogen mustard and acrolein. Acrolein upregulates reactive oxygen species resulting in damage to the urothelial epithelium (1, 10). High doses of cyclophosphamide may lead to accumulation of acrolein in the bladder. This accumulation may cause hematuria, dysuria, urinary frequency, and urinary urgency known as hemorrhagic cystitis (HC) (10–12). There are several methods to prevent HC including hyperhydration, mesna, and continuous bladder irrigation (CBI), but the most effective preventative strategy has not been determined (13–17). Hyperhydration utilizes 2-3 L/m2/day of intravenous fluids with or without diuretics (13, 15), which could potentially lead to fluid overload (FO).

Currently, there is no standardized grading system for FO. In 2017, Rondón et al. proposed a grading system for FO in allogeneic HCT patients (18). Their system graded FO severity from one to four using factors such as percent weight gain from baseline, diuretic use, and organ dysfunction. Using this grading, they compared the prevalence and severity of FO in haploidentical HCT patients who received fludarabine-melphalan (FM) based conditioning to match related or unrelated donor (MRD or MUD) HCT patients who received busulfan-fludarabine (FB) based conditioning. Their results showed that grade 2-4 FO in both cohorts was associated with higher non-relapse mortality (NRM) and worse overall survival (OS) (18). However, this study did not describe the HC prevention strategy used in the PTCy cohort, and FO was diagnosed between the day of admission and Day 30 post-HC. This time frame does not focus on the early phase of HCT when FO can be expected to occur from PTCy and hyperhydration.

In this single center retrospective chart review, we sought to study the prevalence of FO due to hyperhydration given prior to PTCy and the correlation between FO and HCT outcomes. To our knowledge this is the first report to describe the incidence of FO, NRM, and survival outcomes in patients who received preventative hyperhydration with PTCy.

## 2 Methods

### 2.1 Patients and eligibility

This study was approved by the City of Hope (COH) National Medical Center Institutional Review Board. We retrospectively identified a consecutive case series of 275 first time allogeneic HCT patients who received PTCy for GvHD prevention at COH from July 2009 to December 2018. All patients received PTCy along with hydration and mesna according to the COH standard operating procedures (SOP) as described below. These patients also received supportive care for the prevention of veno-occlusive disease/sinusoidal obstructive syndrome (VOD/SOS) and antimicrobial prophylaxis according to the COH SOP. Patients were included in the study if they received a full course of PTCy with hydration and mesna, a calcineurin inhibitor (CI), and mycophenolate mofetil (MMF) as GvHD prophylaxis for their first HCT and had adequate baseline organ function within 30 days of HCT defined by the following criteria: renal function (serum creatinine ≤2 times the upper limit of normal (ULN) or creatinine clearance of >60 mL/min/1.73 m^2^), liver function (total bilirubin ≤2 times the ULN and AST/ALT ≤2.5 times the ULN), cardiac function [LVEF >50% or an appropriate increase in ejection fraction (EF) with exercise in patients with EF <50% and clearance from cardiology], and pulmonary function (FVC and DLCO >50% of predicted). Patients were excluded if they had no recorded weights during their HCT, had documented confounding factors such as infection-related fluid accumulation or nephrotoxicity affecting fluid status during the FO grading period, or early evidence of cardiotoxicity or nephrotoxicity attributed to conditioning.

### 2.2 Conditioning regimens

Conditioning regimens were selected based on patient’s age, comorbidities, disease type, donor type, and disease status at HCT. Myeloablative (MAC) regimens included total body irradiation (TBI)-based on when TBI dose was >800 cGy (e.g., fludarabine/fractionated TBI and fludarabine/cyclophosphamide/total marrow and lymph node irradiation [TMLI]) and non-TBI-based regimens (e.g., busulfan/fludarabine and busulfan/fludarabine/cyclophosphamide). Reduced intensity/non-myeloablative conditioning (RIC/NMA) included fludarabine/cyclophosphamide/TBI, fludarabine/melphalan, and fludarabine/melphalan/TBI.

### 2.3 GvHD regimen and supportive care

PTCy (50 mg/kg) was administered on Days 3 and 4 of alloHCT with hyperhydration and mesna. The hydration consisted of a sodium chloride-base with electrolytes and furosemide additives, administered at a rate of 250 mL/hour for adults and 125 mL/m^2^/hour for pediatric patients. The hydration began 4 hours prior to the start of cyclophosphamide administration and continued until 24 hours after the completion of the second cyclophosphamide infusion. Mesna was started with the first cyclophosphamide infusion; 20 mg/kg was administered every 15 minutes for 16 doses. PTCy was given in combination with tacrolimus (TAC) (0.02-0.03 mg/kg) or flat dose (1 mg), selected per discretion of the treating physician or based on clinical trial, and mycophenolate mofetil (MMF) (15 mg/kg or 1000 mg maximum three times per day) both starting on Day 5 post-HCT through day 90 or through day 35 in the absence of severe GvHD. The TAC and MMF components of the GvHD regimen were modified based on patient specific factors.

### 2.4 Criteria for fluid overload and grading system

There is no standardized grading system for FO. Our grading criteria was adapted and modified from the grading criteria proposed by Rondón et al. (18) (**Table 1**). FO was scored using the following criteria: grade 0 FO was defined as no weight gain, edema, or diuretic therapy, grade 1 FO was defined as weight gain <10% from baseline and asymptomatic or mild edema, which may require occasional diuretic therapy defined as use of a diuretic on at least two days in the time period or a decrease in intravenous fluid replacement; grade 2 FO was defined as possible symptomatic fluid retention with or without weight gain (≥10% to <20% from baseline), but requires ongoing diuretic therapy; grade 3 was defined as FO that does not respond to diuretic therapy with possible weight gain ≥20% from baseline or possible renal, pulmonary, or cardiac dysfunction; grade 4 was defined as FO with progressive dysfunction of more than one organ system or requiring intensive care (18).

**Table 1:** FO grading system adapted from Rondón et al. (18).

Grade	Weight Gain	Symptoms	Diuresis
**1**	<10% from baseline	Asymptomatic or mild edema	Possible diuretic therapy or a decrease in intravenous fluid replacement
**2**	With or without weight gain (≥10% to <20% from baseline)	Possible symptomatic fluid retention	Requiring ongoing diuretic therapy
**3**	With or without weight gain (≥20% from baseline)	Possible renal, pulmonary, or cardiac dysfunction requiring further treatment	Not responding to diuretic therapy
**4**	—	Progressive dysfunction of more than 1 organ system or requiring intensive care	—

Grey = Required. Light grey = May be present in patients.

Hyperhydration was administered on Days 3 to 5 of alloHCT, and the Day 3 to Day 8 (D3 to D8) period was selected to determine the direct effect of hyperhydration on fluid status and HCT outcomes. We collected the maximum recorded weight during D3 to D8 from baseline. We used the pre-transplant weight as baseline weight. FO grade reported for each patient was the highest grade reported from D3 to D8.

### 2.5 Endpoints and definitions

The primary endpoints were prevalence and severity of FO and its impact on non-relapse mortality (NRM). Secondary endpoints included the impact of FO on relapse rate (RR), disease-free survival (DFS), overall survival (OS), incidence of hemorrhagic cystitis (HC), neutrophil engraftment, platelet engraftment, acute GvHD, and chronic GvHD.

NRM was defined as time from transplant to death from any cause without evidence of relapse. Time to relapse (TRR) was defined as time from transplant to the first observation of disease relapse/progression. NRM and TTR were competing risk events and were censored at last follow-up if patients were alive and free of relapse. DFS was defined as time from HCT to first observation of disease relapse or death from any cause without evidence of disease. DFS was censored at last follow-up if patients remained alive and disease-free. OS was defined as time from HCT to death from any cause and was censored at last follow-up if the patient was alive. HC was graded according to criteria for cystitis noninfective published in version 5.0 of the Common Terminology Criteria for Adverse Events (CTCAE). Time to neutrophil engraftment was measured from HCT to the first of 3 consecutive days with neutrophil count greater than 0.5 x10^9^/L. Time to platelet engraftment was measured from HCT to the first day of platelet count greater than 20 x10^9^/L. NRM was the completing risk event for both time to neutrophil engraftment and platelet engraftment. Documented/biopsy proven acute GvHD was graded according to the Glucksberg scale. Time to acute GvHD was measured from HCT to document/biopsy proven acute GVHD onset date within day +100 post HCT. Time to acute GvHD was censored at day + 100 if patients did not develop grade II-IV acute GvHD by day +100, or the last follow-up, whichever came first. Relapse and NRM that occurred prior to day +100 were competing risk events for acute GvHD. Chronic GvHD was scored as limited or extensive chronic GvHD according to the Seattle criteria. Time to chronic GvHD was measured from HCT to the documented/biopsy proven chronic GVHD onset date or censored at the last follow-up if patients did not develop chronic GvHD. Relapse and NRM were the competing risk events for chronic GvHD.

After FO occurred, renal dysfunction was identified using the physician’s diagnosis in the documentation based on increase in serum creatinine from baseline or need for hemodialysis. Cardiac dysfunction was defined by initiation of vasopressor support or echo showing a significant drop in EF. Intensive care was defined as requiring admission to the intensive care unit (ICU) for multi-organ dysfunction excluding patients who only transferred to ICU for the sole purpose of hemodialysis.

### 2.6 Statistical analysis

The baseline patient, disease, and transplant characteristics were analyzed using descriptive statistics. The differences in the baseline characteristics and transplant-related complications by FO grade were examined using Kruskal-Wallis Test for continuous variables and χ^2^ test or Fisher’s exact test for categorical variables. The landmark analysis was used to examine the associations between FO grade and transplant outcomes to avoid immortal-time bias. Patients who died within Day + 8 post HCT were excluded. Kaplan-Meier curves and log-rank tests were used for OS and DFS in the univariate analysis. Cumulative incidence curves and Gray’s tests were used for NRM, TTR, and other competing risks event outcomes in the univariate analysis. Multivariable Cox or Fine and Gray regression models were used to investigate the independent effects of FO on the endpoints. Stepwise regression approach was used to choose baseline variables that were significantly associated with an endpoint at 0.1 level to be included in the final multivariable regression models. Statistical analyses were performed using SAS 9.4 (SAS Institute, Cary, NC). *P* values were two-sided at a 0.05 significance level.

## 3 Results

### 3.1 Identification and patient characteristics

**Figure 1:**
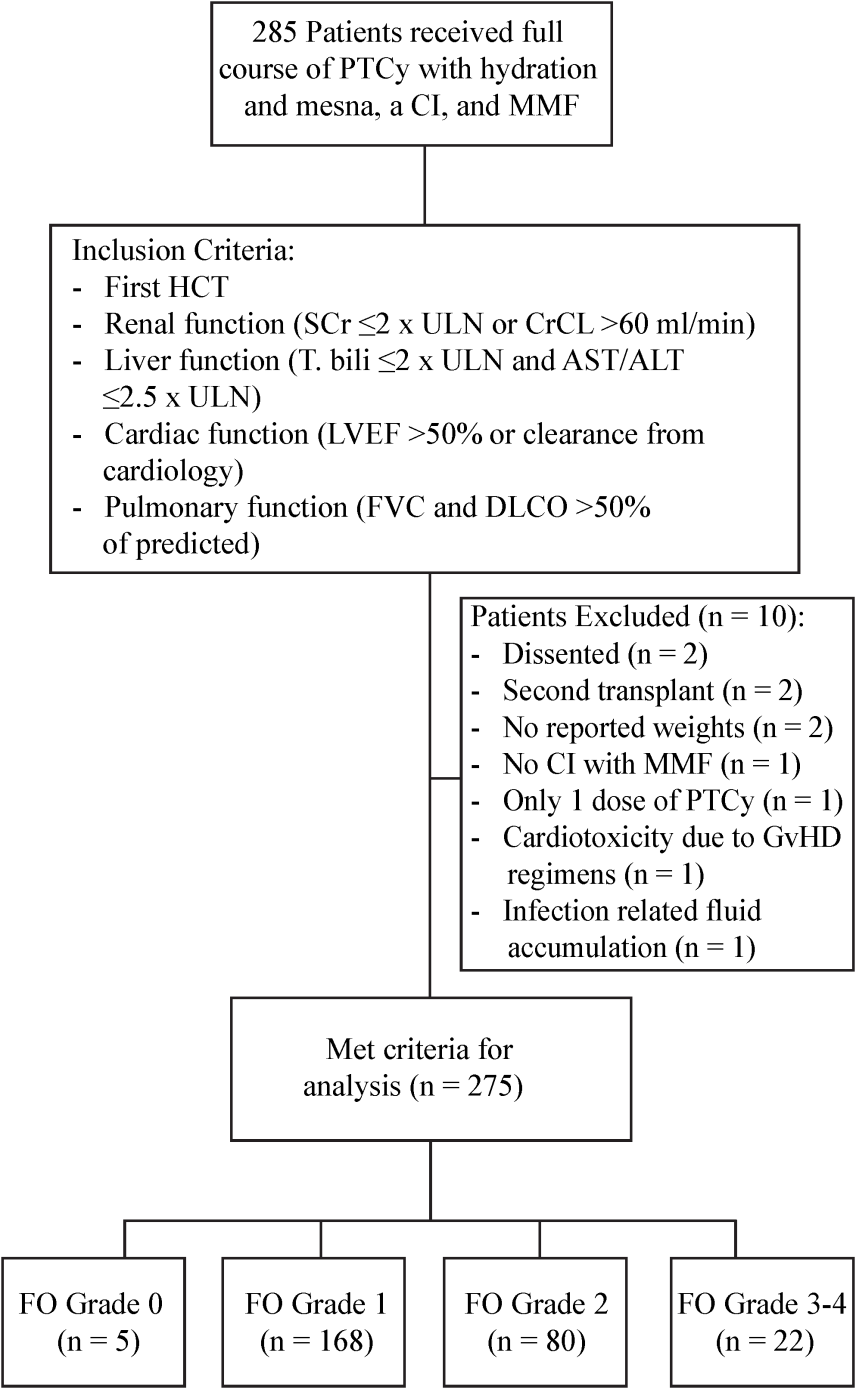
Patient identification.

The details of patient identification are included in **Figure 1**. Patient and HCT characteristics are detailed in **Table 2**. Briefly, at the time of HCT, the median age was 47 years (range=4-75), and 58.5% of patients (n=161) were male. 40.7% of patients (n=112) had a HCT comorbidity index (HCT-CI) of 3 or more. The primary diagnosis for most patients was acute leukemia (60%). Disease risk index (DRI) was high/very high in 34.2% patients. Patients received HCT from haploidentical in 76.4%, mismatched unrelated in 16%, and from matched related or unrelated donor in 7.6%. The majority of patients (80%) received peripheral blood stem cells (PBSC), and 44% of patients received MAC regimen.

**Table 2:** Patient characteristics.

	FO D3 to D8				
	Grade 0-1 (N=173)	Grade 2 (N=80)	Grade 3-4 (N=22)	Total (N=275)	P value
Age at HCT, years					0.0005
Median (Range)	44 (4-71)	58 (4-75)	59 (8-71)	47 (4-75)	
Recipient sex					0.88
Male	103 (59.5%)	45 (56.3%)	13 (59.1%)	161 (58.5%)	
Female	70 (40.5%)	35 (43.8%)	9 (40.9%)	114 (41.5%)	
Female donor to male recipient					1.00
Yes	32 (18.5%)	15 (18.8%)	4 (18.2%)	51 (18.5%)	
No	141 (81.5%)	65 (81.3%)	18 (81.8%)	224 (81.5%)	
Donor age					0.86
Median (Range)	31 (10-65)	33 (13-68)	30 (14-57)	32 (10-68)	
Primary diagnosis					0.03
AML	72 (41.6%)	23 (28.8%)	9 (40.9%)	104 (37.8%)	
ALL	39 (22.5%)	19 (23.8%)	3 (13.6%)	61 (22.2%)	
MDS/CML/MPN	23 (13.3%)	21 (26.3%)	7 (31.8%)	51 (18.5%)	
Lymphoma/MM	26 (15%)	8 (10%)	0 (0%)	34 (12.4%)	
Non-Malignant	13 (7.5%)	9 (11.3%)	3 (13.6%)	25 (9.1%)	
DRI					0.14
Low	34 (19.7%)	13 (16.3%)	1 (4.5%)	48 (17.5%)	
Intermediate	72 (41.6%)	31 (38.8%)	5 (22.7%)	108 (39.3%)	
High/Very High	54 (31.2%)	27 (33.8%)	13 (59.1%)	94 (34.2%)	
Non-malignant	13 (7.5%)	9 (11.3%)	3 (13.6%)	25 (9.1%)	
Karnofsky performance status %					0.21
80-100	154 (89%)	65 (81.3%)	18 (81.8%)	237 (86.2%)	
≤70	19 (11%)	15 (18.8%)	4 (18.2%)	38 (13.8%)	
HCT comorbidity index					0.68
0	45 (26%)	24 (30%)	5 (22.7%)	74 (26.9%)	
1-2	61 (35.3%)	21 (26.3%)	7 (31.8%)	89 (32.4%)	
≥3	67 (38.7%)	35 (43.8%)	10 (45.5%)	112 (40.7%)	
Donor Type					0.99
Haploidentical	130 (75.1%)	62 (77.5%)	18 (81.8%)	210 (76.4%)	
Mismatched unrelated	29 (16.8%)	12 (15%)	3 (13.6%)	44 (16%)	
Matched related/unrelated	14 (8.1%)	6 (7.5%)	1 (4.5%)	21 (7.6%)	
Stem Cell Source					0.013
Bone Marrow	44 (25.4%)	8 (10%)	3 (13.6%)	55 (20%)	
Peripheral Blood Stem Cells	129 (74.6%)	72 (90%)	19 (86.4%)	220 (80%)	
ABO blood group compatibility					0.76
ABO compatible	116 (67.1%)	53 (66.3%)	15 (68.2%)	184 (66.9%)	
Minor mismatch (donor is O)	21 (12.1%)	11 (13.8%)	5 (22.7%)	37 (13.5%)	
Major mismatch (Recipient is O)	22 (12.7%)	11 (13.8%)	2 (9.1%)	35 (12.7%)	
Bidirectional (None are O)	14 (8.1%)	5 (6.3%)	0 (0%)	19 (6.9%)	
Donor/Recipient CMV serostatus					0.029
D-/R-	21 (12.1%)	2 (2.5%)	2 (9.1%)	25 (9.1%)	
D-/R+	41 (23.7%)	24 (30%)	1 (4.5%)	66 (24%)	
D+/R-	12 (6.9%)	9 (11.3%)	1 (4.5%)	22 (8%)	
D+/R+	99 (57.2%)	45 (56.3%)	18 (81.8%)	162 (58.9%)	
Conditioning Regimen					0.002
MAC	90 (52%)	24 (30%)	7 (31.8%)	121 (44%)	
RIC	83 (48%)	56 (70%)	15 (68.2%)	154 (56%)	
GVHD prophylaxis					
SIRO/CY/MMF	0 (0%)	0 (0%)	1 (4.5%)	1 (0.4%)	
TAC/CY	6 (3.5%)	1 (1.3%)	0 (0%)	7 (2.5%)	
TAC/CY/MMF	167 (96.5%)	79 (98.8%)	21 (95.5%)	267 (97.1%)	
HCT Period					0.25
2009-2015	45 (26%)	15 (18.8%)	3 (13.6%)	63 (22.9%)	
2016-2018	128 (74%)	65 (81.3%)	19 (86.4%)	212 (77.1%)	

MAC, myeloablative conditioning; RIC, reduced intensity conditioning; SIRO, sirolimus; CY, cyclophosphamide, TAC, tacrolimus; MMF, mycophenolate mofetil.

### 3.2 Prevalence and severity of fluid overload

During D3 to D8 after HCT, 270 patients (98%) were diagnosed with mild to severe FO. Of the total 275 patients, 5 patients (2% of total) did not develop FO (grade 0), 168 patients (61%) experienced mild (grade 1), 80 (29%) had moderate (grade 2) FO, and 22 (8%) experienced severe (grade 3-4) FO. Patients with severe (grade 3-4) FO (n=22) were older (median 59 years; p= 0.0005), more likely to have AML (40.9%, p=0.03), and more likely to undergo lower intensity regimen (RIC/NMA in 68.2%, p=0.002) and PBSC grafts (in 86.4%, p=0.013) compared to patients with mild (grade 0-1) to moderate FO (grade 2).

### 3.3 Engraftment and GVHD

Ninety-two percent of patients had neutrophil engraftment by day 28. This was lower in patients with severe FO (63.6%; 95% CI: 0.39-0.81) compared to patients with mild FO (97.7%; 95% CI: 0.94-0.99) or moderate FO (91.3%; 95% CI: 0.82-0.96). Moreover, median time to neutrophil engraftment was longer in patients with severe FO compared to mild FO (22 days vs 16 days, 95% CI: 16-17, p<0.001) and moderate FO (22 days vs 18 days, 95% CI: 17-19, p< 0.001).

Platelet engraftment was achieved in 78.5% of patients by day 42 post-HCT. This was also lower in patients with severe FO achieved platelet engraftment (13.6%; 95% CI: 0.046-0.404, p< 0.001) compared to patients with mild FO (87.9%; 95% CI: 0.83-0.93, p<0.001) or moderate FO (76.2%; 95% CI: 0.67-0.86, p<0.001). In multivariate analysis, severe FO was associated with worse neutrophil (HR = 0.34, 95% CI: 0.21-0.57, p<0.001) and platelet engraftment (HR = 0.13, 95% CI: 0.07-0.23, p<0.001).

Day 100 cumulative incidence (CI) of grade II-IV and III/IV acute GvHD (aGvHD) were 43.4% (95% CI: 0.36-0.51) and 11.6% (95% CI: 0.07-0.17) for patients with mild FO, 42.5% (95% CI: 0.32-0.53) and 16.3% (95% CI: 0.09-0.25) with moderate FO, and 50% (95% CI: 0.27-0.69) and 27.3% (95% CI: 0.11-0.47) with severe FO. In multivariate analysis (**Supplementary Table 1**), severity of FO was not associated with grade II-IV or grade III-IV aGvHD. However, PBSC graft (p=0.031) was associated with higher CI of grade II-IV aGvHD.

The CI of any and extensive cGvHD at 12 months was 45.9% (95% CI: 0.38-0.53) and 33.7% (95% CI: 0.27-0.41) for patients with mild FO, 36.3% (95% CI: 0.26-0.47) and 27.5% (95% CI: 0.18-0.38) with moderate FO, and 9.1% and 9.1% (95% CI: 0.13-0.26) with severe FO. In multivariate analysis, grade 0-1 FO (P = 0.014) was associated with higher incidence of any grade cGvHD, but any grade FO was not associated with extensive cGVHD. Female donor to male recipient (F to M) (p=0.004), donor age ≥ 35 (p=0.047) and donor type (p=0.038) were associated with any grade cGvHD, while female patients (p=0.044), F to M (p=0.013), donor age ≥ 35 (p=0.019) and MAC conditioning (p=0.041) were associated with extensive cGvHD.

### 3.4 Transplant-related complications

For patient with FO, transplant related complications within 100 days of HCT included hemodialysis in 10 (3.6%) patients, ICU admissions for 28 (10.2%) patients, VOD/SOS diagnosed in 7 (2.5%) patients, and HC in 81 (29.5%) patients (grade 1: 68 [24.4%] and grade 2: 14 patients [5.1%]). Despite small numbers, VOD (18.2% vs 1.2%, p<0.001), HC (22.7% vs 3.5%, p<0.001), and ICU admission (81.8% vs 4%, p<0.001) were all associated with severe FO compared to mild-moderate FO.

### 3.5 NRM and relapse rate

**Figure 2:**
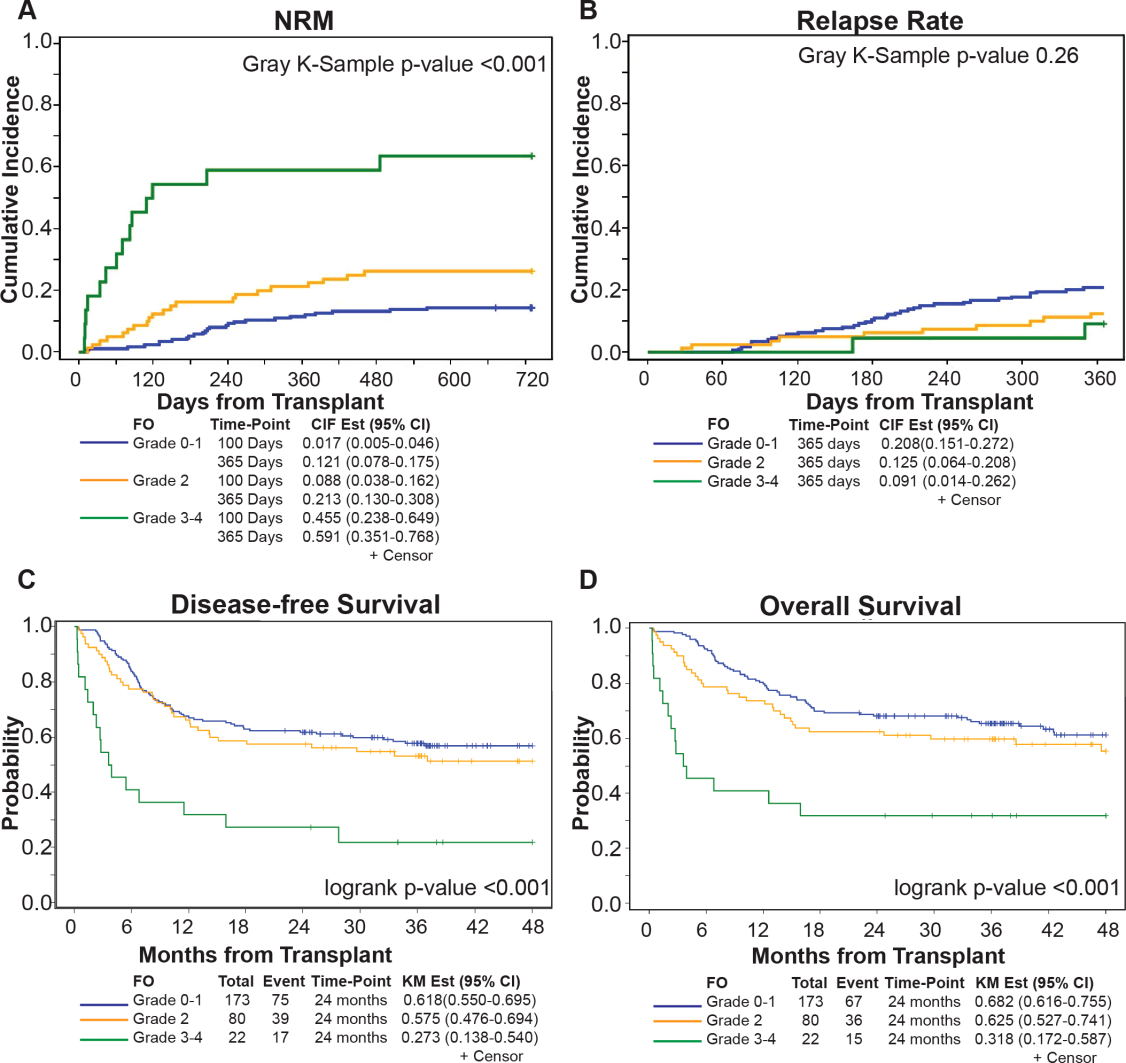
Outcomes comparing Grade 0-1, 2, and 3-4 FO patients. Cumulative incidence of **(A)** Non-relapse mortality. **(B)** Relapse. Kaplan Meier curves of **(C)** Disease-free survival, **(D)** Overall survival.

**Figure 3:**
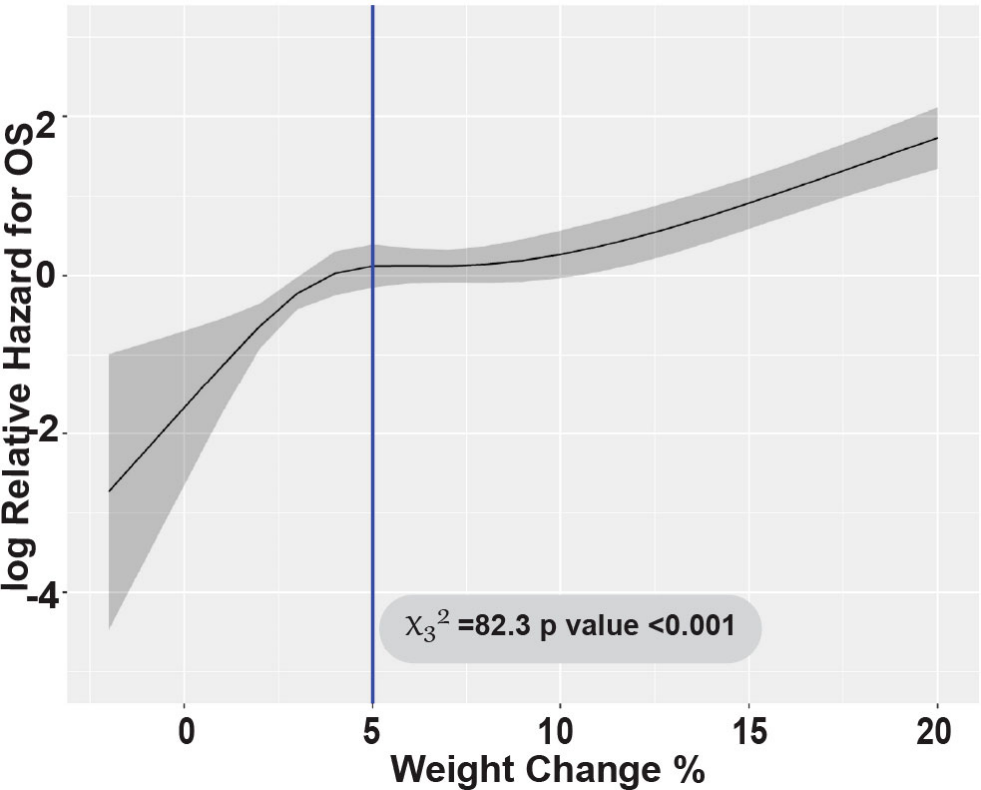
Linear association between weight gain and overall survival.

With a median follow-up of 23.9 months (range: 4.1-64.4), NRM at 100 days post-HCT was significantly higher in patients with severe FO (45.5%) compared to mild-to-moderate FO patients (grade 0-1: 1.7% and grade 2: 8.8%, p<0.001 respectively). This difference persisted at 1 year with severe FO (59.1%) compared to mild-to-moderate FO patients with grade 0-1 and 2 (59.1% vs 12.1% and 21.3%, p<0.001, respectively) (**Figure 2A**). Most of patients with severe FO died of infection and organ dysfunction (73.3% and 20%) compared to (41.8 and 4.5%, p=0.019, respectively) in mild to moderate FO. In multivariate analysis, severe FO was associated with significantly higher NRM (HR = 5.67, 95% CI: 2.79-11.51, p<0.001). Weight gain was tested as continuous variable; every 5% increase in weight was also associated with significantly higher NRM (HR = 1.91, 95% CI: 1.64-2.23, p<0.001) (**Figure 3**). FO did not impact relapse rate at 2 years (grade 0-1 = 23.7%, grade 2 = 16.2%, grade 3-4 = 9.1%, p=0.26) (**Figure 2B**). In a multivariate analysis, intermediate to high DRI (HR = 2.29, 95% CI: 0.99-5.31, p<0.001) and with BM graft (HR = 2.73, 95% CI: 1.60-4.65, p<0.001) were associated with higher relapse. Full multivariable analyses of NRM and relapse are in **Supplementary Table 2**.

### 3.6 Survival outcomes

At 2 years post-HCT, the DFS for patients with severe FO was significantly lower compared to patients who had mild-to-moderate (grade 0-1 and 2) FO (27.3% vs 61.8% and 57.5%, p<0.001, respectively) (**Figure 2C**). This association was validated in multivariable analysis; severe FO (HR=4.05, 95% CI: 2.37-6.92, p<0.001) was associated with a lower DFS. Similarly, OS at 2 years post-HCT for severe FO patients was significantly lower compared to mild-to-moderate FO patients (31.8% vs 68.2% and 62.5%, p<0.001, respectively) (**Figure 2D**). This was also confirmed by multivariable analysis; severe FO was associated with lower OS compared to mild-moderate FO (HR=3.48, 95% CI: 1.94-6.24, p<0.001). Additionally, each 5% increase in weight was also associated with significantly lower two-year DFS and OS (HR=1.77, 95% CI: 1.53-2.04, p<0.001 and HR=1.89, 95% CI: 1.64-2.19, p<0.001, respectively). The Wald’s test revalidated this by showing a linear association with the log relative hazard for OS with weight gain ≥5% during the study period (p<0.001) (**Figure 2**).

In multivariate analysis, age (p=0.009), intermediate to high DRI (p<0.001), KPS ≤ 80 (p<0.001), HCT-CI ≥3 (p=0.049), RIC/NMA Conditioning (p=0.023), and BM graft (p=0.045) were associated with lower DFS, while age >60 years (p<0.003), intermediate to high DRI (p<0.001) and KPS ≤80 (p=0.02) were associated with lower OS. Full multivariable analyses of survival outcomes are in **Supplementary Table 3**.

## 4 Discussion

The use of PTCy for GvHD prophylaxis in HCT patients has increased access to potentially life-saving therapy for thousands of patients without matched sibling or unrelated donors, particularly in underrepresented, ethnic groups (1–3, 5, 6). In addition, PTCy in combination with tacrolimus and mycophenolate mofetil (MMF) has been recently shown to increase the probability of 1-year of GVHD-free, relapse-free survival (GRFS) for closely matched patients receiving RIC (4, 8). This has led to a surge in use of PTCy which will likely increase the incidence of PTCy-associated HC. Furthermore, there is no optimal strategy for HC prevention (10–13) after PTCy administration in HCT recipients. Hyperhydration is an effective method of HC prevention but can also lead to complications of FO, which can contribute to multi-organ dysfunction early in the HCT (19).

Our results showed that FO is common complication with hyperhydration from D3 to D8. This FO incidence is higher than the results reported in previous studies, which may be a result of either a higher total amount of fluid received by our patients or due to restricting the study time frame to D3 to D8 to capture the immediate effect of hyperhydration. Although there is a higher prevalence of FO, the clinical consequences of FO are consistent with these studies (18, 20–23) where severe FO had significantly higher NRM and worse survival outcomes. Our results reiterate the need for a standardized FO grading system for HCT patients because it can assist in early recognition of FO and intervention that can avoid FO and the poor associated clinical outcomes (18).

Our study and others highlight that maintaining fluid balance is critical in the early phase after HCT and suggest that the severity FO is associated with organ dysfunction, which necessitates the need for intensive intervention. This need was shown in the patients with severe FO who had a higher incidence transplant-related complications such as hemodialysis, vasopressors, and/or ICU admissions needed to manage FO-related organ dysfunction. Our study also brings awareness to the fact that hyperhydration for HC prevention might not fit all patients, and that individualization of care is necessary to mitigate this risk such as utilizing continuous bladder irrigation (CBI) for patients with risk factors associated with FO (17, 24). For instance, our results show that advanced age, lower KPS, and high DRI are associated with lower survival outcomes, which suggests that these patients may need to utilize alternative strategies for HC prevention to prevent development of severe FO. Another approach is to limit hydration by applying a frequent intermittent bolus administration of mesna with at least 100% of the PTCy dose as an effective method for preventing HC (25). Our study emphasizes the need for more comparative studies to determine the optimal preventative strategy for PTCy associated HC and risk factors involved in each is warranted.

In 2003, Mank et al. established monitoring weight to determine patient’s fluid balance is a safe method for patients receiving hyperhydration (26). In our study we found strong linear associations between weight gain from baseline and worse NRM, DFS, and OS. For every 5% increase in weight correlated with 2-fold increase in NRM and 1.75-fold decrease in survival. While our study was specific to the peri-PTCy phase, it re-substantiates the same conclusion reached by Shiomura et al., that weight gain greater than 5% is associated with poor OS in HCT patients (23). During the early period of HCT, close monitoring of weight and early initiation of interventions, such as reduction of fluid replacement or start of diuretics may prevent high grade FO and its negative clinical outcomes.

The main limitation of our study is the nature of single center retrospective review. Although baseline organ dysfunction was ruled out by reviewing pre-HCT values and factors that could affect fluid status such as VOD/SOS, and kidney or cardiac dysfunction due to conditioning or other etiologies, our evaluation is limited by information provided in the form of medical chart review. Two-time points for renal and cardiac function were assessed through clinical review of charts (including 4–6-week pre HCT and day 3 baseline) allowed accuracy to exclude any pre-existing or conditioning-related organ dysfunction that could cause or overlap the clinical picture of FO. Due to the retrospective manner of this study, it is not possible to definitively exclude the direct effect of PTCy on cardiac function although we have excluded all patients who had abnormal echo results within this timeframe. Moreover, patients undergoing PTCy-based GvHD prophylaxis are at risk of CRS thus the possibility of overlap between FO and CRS makes it difficult to exclude mutual relationship.

In summary, patients who received hyperhydration to prevent PTCy associated HC had a high incidence of FO. Consistent with other studies exploring FO in HCT patients, FO is one of the important predictors of higher NRM and worse survival outcomes. Our study showed that increase in weight after PTCy initiation is strongly associated with worse NRM, DFS, and OS. As FO with weight gain ≥5% is associated with worse outcomes, this marker could be used as an indicator to initiate early interventions to prevent the development of high-grade FO and will become increasingly relevant with broadening indications and uses of PTCy.

## Data Availability

The original contributions presented in the study are included in the article/**Supplementary Material**. Further inquiries can be directed to the corresponding author.
