# Supplementary material for: Impact of hyperhydration on fluid overload and hematopoietic cell transplant after post-transplant cyclophosphamide-based graft-versus-host-disease prophylaxis
Source: Front Immunol. 2025 Feb 20;16:1543099. doi: 10.3389/fimmu.2025.1543099 (PMC11882550; doi:10.3389/fimmu.2025.1543099)
Supplement: Supplementary file 1 [file Table1.docx]

Supplementary Material

Supplementary Table 1: Multivariate Analysis for Engraftment and GvHD

|  | | | | | ***Neutrophil Engraftment*** | | | | | ***Platelet Engraftment*** | | | | |
| --- | --- | --- | --- | --- | --- | --- | --- | --- | --- | --- | --- | --- | --- | --- |
| ***Variable*** |  | ***N*** | ***28 Day (95%CI)**** | ***HR (95%CI)**** | | ***Gray P**** | ***Adjusted HR (95%CI)* †** | ***FG test P*†** | ***42 Day (95%CI)**** | | ***HR (95%CI)**** | ***Gray P**** | ***Adjusted HR (95%CI)* †** | ***FG test P*†** |
| Age, years | ≤17 | 26 | 0.885(0.654,0.965) | Reference | | **0.043** | Reference | 0.10 | 0.731(0.504,0.866) | | Reference | **0.008** | Reference | 0.16 |
|  | 18-39 | 81 | 0.963(0.876,0.989) | 1.06(0.63,1.79) | |  | 0.92(0.52,1.61) |  | 0.864(0.765,0.924) | | 1.24(0.72,2.14) |  | 1.19(0.67,2.13) |  |
|  | 40-59 | 90 | 0.944(0.866,0.978) | 0.97(0.59,1.62) | |  | 0.84(0.46,1.52) |  | 0.778(0.675,0.851) | | 0.98(0.57,1.69) |  | 1.02(0.55,1.90) |  |
|  | ≥60 | 78 | 0.897(0.801,0.948) | 0.67(0.40,1.12) | |  | 0.60(0.31,1.14) |  | 0.731(0.616,0.816) | | 0.69(0.40,1.19) |  | 0.76(0.39,1.49) |  |
|  | Per 5 yr |  |  | 0.96(0.93,0.99) | | **0.010** | 0.96(0.92,1.00) | 0.054 |  | | 0.95(0.92,0.98) | **0.004** | 0.96(0.91,1.00) | 0.073 |
| Sex | M | 161 | 0.944(0.894,0.971) | Reference | | 0.66 | Reference | 0.58 | 0.783(0.710,0.839) | | Reference | 0.32 | Reference | 0.43 |
|  | F | 114 | 0.912(0.841,0.953) | 1.06(0.84,1.33) | |  | 1.07(0.84,1.36) |  | 0.789(0.702,0.854) | | 1.13(0.87,1.47) |  | 1.11(0.85,1.46) |  |
| KPS | ≥80 | 237 | 0.932(0.892,0.958) | Reference | | 0.68 | Reference | 0.89 | 0.797(0.740,0.843) | | Reference | 0.17 | Reference | 0.43 |
|  | ≤70 | 38 | 0.921(0.753,0.976) | 0.93(0.68,1.28) | |  | 0.97(0.68,1.40) |  | 0.711(0.533,0.830) | | 0.76(0.51,1.13) |  | 0.85(0.56,1.28) |  |
| HCTCI | 0 | 74 | 0.919(0.822,0.964) | Reference | | 0.061 | Reference | 0.12 | 0.770(0.654,0.852) | | Reference | 0.43 | Reference | 0.56 |
|  | 1-2 | 89 | 0.978(0.896,0.995) | 1.23(0.92,1.63) | |  | 1.12(0.83,1.52) |  | 0.798(0.697,0.868) | | 1.12(0.79,1.59) |  | 1.02(0.71,1.47) |  |
|  | ≥3 | 112 | 0.902(0.827,0.945) | 0.93(0.70,1.24) | |  | 0.87(0.63,1.18) |  | 0.786(0.696,0.852) | | 0.94(0.69,1.28) |  | 0.88(0.64,1.22) |  |
|  | Per 1 |  |  | 0.98(0.92,1.03) | | 0.43 | 0.98(0.92,1.04) | 0.46 |  | | 0.97(0.91,1.03) | 0.29 | 0.96(0.90,1.03) | 0.31 |
| DRI | Low | 48 | 0.979(0.769,0.998) | Reference | | 0.11 | Reference | 0.22 | 0.854(0.711,0.930) | | Reference | 0.084 | Reference | 0.33 |
|  | Int-high | 202 | 0.916(0.867,0.947) | 0.78(0.61,1.01) | |  | 0.79(0.60,1.06) |  | 0.762(0.697,0.815) | | 0.75(0.53,1.04) |  | 0.77(0.55,1.08) |  |
|  | Non-malig | 25 | 0.960(0.598,0.997) | 0.97(0.61,1.56) | |  | 1.01(0.59,1.72) |  | 0.840(0.606,0.941) | | 0.99(0.60,1.66) |  | 0.83(0.45,1.55) |  |
| Conditioning | MAC | 121 | 0.950(0.889,0.978) | Reference | | **<0.001** | Reference | **0.036** | 0.843(0.764,0.897) | | Reference | **<0.001** | Reference | 0.063 |
|  | RIC/NMA | 154 | 0.916(0.858,0.951) | 0.66(0.52,0.83) | |  | 0.74(0.56,0.98) |  | 0.740(0.663,0.803) | | 0.63(0.49,0.81) |  | 0.73(0.53,1.02) |  |
| Graft | PBSC | 220 | 0.932(0.889,0.959) | Reference | | 0.23 | Reference | 0.050 | 0.791(0.731,0.839) | | Reference | 0.52 | Reference | 0.40 |
|  | BM | 55 | 0.927(0.807,0.974) | 0.82(0.65,1.03) | |  | 0.74(0.54,1.00) |  | 0.764(0.624,0.857) | | 0.90(0.67,1.21) |  | 0.86(0.60,1.23) |  |
| F to M | No | 224 | 0.929(0.885,0.956) | Reference | | 0.72 | Reference | 0.56 | 0.777(0.716,0.826) | | Reference | 0.51 | Reference | 0.55 |
|  | Yes | 51 | 0.941(0.808,0.983) | 0.94(0.73,1.22) | |  | 0.92(0.71,1.21) |  | 0.824(0.682,0.906) | | 1.11(0.85,1.45) |  | 1.10(0.81,1.47) |  |
| Donor age | ≤34 | 157 | 0.924(0.868,0.956) | Reference | | 0.14 | Reference | **0.036** | 0.771(0.696,0.829) | | Reference | 0.14 | Reference | **0.039** |
|  | ≥35 | 118 | 0.941(0.876,0.972) | 1.17(0.94,1.46) | |  | 1.28(1.02,1.60) |  | 0.805(0.720,0.867) | | 1.21(0.94,1.55) |  | 1.31(1.01,1.69) |  |
|  | Per 5 yr |  |  | 1.01(0.96,1.05) | | 0.75 | 1.02(0.98,1.07) | 0.33 |  | | 1.01(0.96,1.07) | 0.60 | 1.03(0.97,1.09) | 0.35 |
| Donor Type | Haplo | 210 | 0.933(0.889,0.960) | Reference | | **0.026** | Reference | **0.008** | 0.767(0.703,0.818) | | Reference | **0.025** | Reference | **0.021** |
|  | MRD/MUD | 21 | 0.952(0.636,0.995) | 1.47(0.97,2.24) | |  | 1.63(1.12,2.36) |  | 0.905(0.612,0.980) | | 1.55(0.98,2.48) |  | 1.66(1.12,2.48) |  |
|  | MMUD | 44 | 0.909(0.761,0.967) | 1.22(0.87,1.72) | |  | 1.40(1.01,1.95) |  | 0.818(0.662,0.907) | | 1.22(0.87,1.71) |  | 1.38(0.95,2.01) |  |
| HCT era | 2009-17 | 176 | 0.926(0.875,0.957) | Reference | | 0.40 | Reference | 0.65 | 0.750(0.679,0.808) | | Reference | **0.013** | Reference | 0.078 |
|  | 2018 | 99 | 0.939(0.866,0.973) | 1.10(0.88,1.38) | |  | 0.94(0.74,1.21) |  | 0.848(0.760,0.906) | | 1.37(1.06,1.77) |  | 1.28(0.97,1.68) |  |
| FO day 3-8 | 0-1 | 173 | 0.977(0.935,0.992) | Reference | | **<0.001** | Reference | **<0.001** | 0.879(0.819,0.920) | | Reference | **<0.001** | Reference | **<0.001** |
|  | 2 | 80 | 0.913(0.821,0.958) | 0.70(0.55,0.90) | |  | 0.73(0.55,0.96) |  | 0.762(0.652,0.842) | | 0.57(0.43,0.76) |  | 0.58(0.42,0.79) |  |
|  | 3-4 | 22 | 0.636(0.388,0.806) | 0.32(0.20,0.53) | |  | 0.34(0.21,0.57) |  | 0.136(0.032,0.315) | | 0.13(0.07,0.24) |  | 0.13(0.07,0.23) |  |
| FO day 30 | 0-1 | 121 | 0.975(0.917,0.993) | Reference | | **<0.001** | Reference | **<0.001** | 0.884(0.811,0.930) | | Reference | **<0.001** | Reference | **<0.001** |
|  | 2 | 132 | 0.939(0.880,0.970) | 0.78(0.62,0.98) | |  | 0.82(0.64,1.04) |  | 0.803(0.724,0.862) | | 0.60(0.46,0.77) |  | 0.61(0.47,0.80) |  |
|  | 3-4 | 22 | 0.636(0.388,0.806) | 0.32(0.19,0.53) | |  | 0.35(0.21,0.58) |  | 0.136(0.032,0.315) | | 0.12(0.06,0.22) |  | 0.12(0.06,0.22) |  |
| Weight Δ | ≤5% | 137 | 0.949(0.893,0.976) | Reference | | **0.001** | Reference | **<0.001** | 0.883(0.815,0.927) | | Reference | **<0.001** | Reference | **<0.001** |
|  | >5-10% | 96 | 0.969(0.895,0.991) | 0.80(0.63,1.02) | |  | 0.81(0.62,1.05) |  | 0.792(0.694,0.861) | | 0.71(0.54,0.93) |  | 0.73(0.54,0.98) |  |
|  | >10% | 42 | 0.786(0.621,0.885) | 0.42(0.31,0.59) | |  | 0.46(0.32,0.66) |  | 0.452(0.296,0.596) | | 0.27(0.18,0.39) |  | 0.29(0.19,0.43) |  |
| Weight Δ | Per 5% |  |  | 0.75(0.68,0.84) | | **<0.001** | 0.78(0.70,0.87) | **<0.001** |  | | 0.60(0.52,0.69) | **<0.001** | 0.61(0.53,0.72) | **<0.001** |

* Based on univariate analysis and Gray’s test

† Based on the multivariable Fine and Gray models. Models on neutrophil engraftment were adjusted for recipient age per 5 years, conditioning, graft source, and donor type. Models on platelet engraftment were adjusted for recipient age per 5 years, conditioning, graft source, donor type, and HCT era.

|  | | | | | ***Grade II-IV aGVHD*** | | | | | ***Grade III-IV aGVHD*** | | | | |
| --- | --- | --- | --- | --- | --- | --- | --- | --- | --- | --- | --- | --- | --- | --- |
| ***Variable*** |  | ***N*** | ***100 days (95%CI)**** | ***HR (95%CI)**** | | ***Gray P**** | ***Adjusted HR (95%CI)* †** | ***FG test P*†** | ***100 days (95%CI)**** | | ***HR (95%CI)**** | ***Gray P**** | ***Adjusted HR (95%CI)* †** | ***FG test P*†** |
| Age, years | ≤17 | 26 | 0.385(0.200,0.567) | Reference | | 0.15 | Reference | 0.18 | 0.115(0.028,0.271) | | Reference | 0.98 | Reference | 0.99 |
|  | 18-39 | 81 | 0.444(0.334,0.549) | 1.26(0.63,2.49) | |  | 1.05(0.51,2.16) |  | 0.148(0.081,0.235) | | 1.29(0.36,4.57) |  | 0.93(0.24,3.61) |  |
|  | 40-59 | 90 | 0.522(0.414,0.620) | 1.54(0.80,2.97) | |  | 1.20(0.59,2.45) |  | 0.144(0.081,0.225) | | 1.26(0.36,4.44) |  | 0.84(0.21,3.29) |  |
|  | ≥60 | 78 | 0.346(0.242,0.452) | 0.92(0.46,1.86) | |  | 0.72(0.34,1.53) |  | 0.141(0.075,0.228) | | 1.23(0.34,4.45) |  | 0.83(0.21,3.24) |  |
|  | Per 5 yr |  |  | 0.98(0.94,1.03) | | 0.45 | 0.96(0.92,1.01) | 0.13 |  | | 1.00(0.92,1.08) | 0.97 | 0.97(0.89,1.06) | 0.54 |
| Sex | M | 161 | 0.460(0.381,0.535) | Reference | | 0.55 | Reference | 0.63 | 0.155(0.104,0.216) | | Reference | 0.49 | Reference | 0.53 |
|  | F | 114 | 0.404(0.313,0.492) | 0.89(0.62,1.28) | |  | 0.91(0.63,1.32) |  | 0.123(0.070,0.191) | | 0.80(0.41,1.53) |  | 0.81(0.42,1.57) |  |
| KPS | ≥80 | 237 | 0.435(0.371,0.497) | Reference | | 0.61 | Reference | 0.62 | 0.135(0.095,0.182) | | Reference | 0.43 | Reference | 0.40 |
|  | ≤70 | 38 | 0.447(0.284,0.598) | 1.13(0.69,1.88) | |  | 1.14(0.68,1.91) |  | 0.184(0.080,0.322) | | 1.39(0.62,3.13) |  | 1.42(0.63,3.20) |  |
| HCTCI | 0 | 74 | 0.378(0.268,0.488) | Reference | | 0.32 | Reference | 0.42 | 0.108(0.050,0.191) | | Reference | 0.59 | Reference | 0.70 |
|  | 1-2 | 89 | 0.483(0.375,0.583) | 1.43(0.90,2.28) | |  | 1.35(0.85,2.17) |  | 0.146(0.082,0.228) | | 1.40(0.58,3.37) |  | 1.28(0.53,3.10) |  |
|  | ≥3 | 112 | 0.438(0.344,0.527) | 1.16(0.74,1.82) | |  | 1.11(0.71,1.74) |  | 0.161(0.100,0.235) | | 1.53(0.67,3.49) |  | 1.42(0.62,3.26) |  |
|  | Per 1 |  |  | 1.02(0.94,1.11) | | 0.62 | 1.01(0.93,1.10) | 0.77 |  | | 1.05(0.91,1.21) | 0.50 | 1.04(0.89,1.20) | 0.63 |
| DRI | Low | 48 | 0.438(0.294,0.573) | Reference | | 0.88 | Reference | 0.93 | 0.083(0.026,0.183) | | Reference | 0.43 | Reference | 0.34 |
|  | Int-high | 202 | 0.441(0.371,0.508) | 1.00(0.63,1.59) | |  | 0.95(0.60,1.52) |  | 0.153(0.108,0.207) | | 1.93(0.69,5.41) |  | 1.81(0.64,5.11) |  |
|  | Non-malig | 25 | 0.400(0.208,0.586) | 0.85(0.41,1.77) | |  | 1.08(0.48,2.40) |  | 0.160(0.049,0.329) | | 1.98(0.51,7.79) |  | 2.76(0.70,10.94) |  |
| Conditioning | MAC | 121 | 0.488(0.395,0.573) | Reference | | 0.10 | Reference | 0.15 | 0.099(0.054,0.160) | | Reference | 0.077 | Reference | 0.054 |
|  | RIC/NMA | 154 | 0.396(0.318,0.473) | 0.74(0.52,1.05) | |  | 0.77(0.54,1.10) |  | 0.175(0.120,0.240) | | 1.83(0.93,3.60) |  | 1.96(0.99,3.89) |  |
| Graft | PBSC | 220 | 0.464(0.396,0.528) | Reference | | **0.035** | Reference | **0.031** | 0.159(0.114,0.211) | | Reference | 0.094 | Reference | 0.10 |
|  | BM | 55 | 0.327(0.207,0.453) | 0.59(0.37,0.95) | |  | 0.59(0.37,0.95) |  | 0.073(0.023,0.162) | | 0.43(0.16,1.19) |  | 0.43(0.16,1.19) |  |
| F to M | No | 224 | 0.455(0.389,0.519) | Reference | | 0.16 | Reference | 0.13 | 0.143(0.101,0.192) | | Reference | 0.88 | Reference | 0.79 |
|  | Yes | 51 | 0.353(0.224,0.484) | 0.73(0.46,1.16) | |  | 0.69(0.43,1.12) |  | 0.137(0.060,0.247) | | 0.94(0.42,2.10) |  | 0.89(0.38,2.07) |  |
| Donor age | ≤34 | 157 | 0.439(0.361,0.516) | Reference | | 0.94 | Reference | 0.91 | 0.146(0.096,0.207) | | Reference | 0.84 | Reference | 0.98 |
|  | ≥35 | 118 | 0.432(0.341,0.520) | 0.98(0.68,1.40) | |  | 1.02(0.71,1.47) |  | 0.136(0.081,0.204) | | 0.94(0.50,1.77) |  | 0.99(0.52,1.88) |  |
|  | Per 5 yr |  |  | 1.00(0.92,1.09) | | 0.99 | 1.01(0.93,1.10) | 0.84 |  | | 1.01(0.86,1.19) | 0.88 | 1.02(0.87,1.21) | 0.79 |
| Donor Type | Haplo | 210 | 0.419(0.352,0.485) | Reference | | **0.028** | Reference | 0.072 | 0.129(0.087,0.178) | | Reference | 0.41 | Reference | 0.62 |
|  | MRD/MUD | 21 | 0.286(0.113,0.487) | 0.64(0.30,1.40) | |  | 0.64(0.29,1.40) |  | 0.143(0.034,0.326) | | 1.15(0.34,3.88) |  | 1.17(0.35,3.92) |  |
|  | MMUD | 44 | 0.591(0.428,0.721) | 1.64(1.07,2.51) | |  | 1.51(0.97,2.33) |  | 0.205(0.100,0.335) | | 1.68(0.79,3.55) |  | 1.48(0.67,3.24) |  |
| HCT era | 2009-17 | 176 | 0.415(0.341,0.487) | Reference | | 0.19 | Reference | 0.30 | 0.142(0.095,0.198) | | Reference | 0.93 | Reference | 0.84 |
|  | 2018 | 99 | 0.475(0.373,0.570) | 1.30(0.90,1.87) | |  | 1.22(0.84,1.76) |  | 0.141(0.081,0.218) | | 1.03(0.53,1.99) |  | 0.93(0.48,1.82) |  |
| FO day 3-8 | 0-1 | 173 | 0.434(0.359,0.506) | Reference | | 0.83 | Reference | 0.93 | 0.116(0.073,0.168) | | Reference | 0.11 | Reference | 0.17 |
|  | 2 | 80 | 0.425(0.315,0.531) | 1.05(0.70,1.57) | |  | 0.98(0.65,1.47) |  | 0.163(0.091,0.252) | | 1.47(0.73,2.95) |  | 1.34(0.67,2.69) |  |
|  | 3-4 | 22 | 0.500(0.270,0.693) | 1.18(0.64,2.19) | |  | 1.12(0.59,2.10) |  | 0.273(0.107,0.471) | | 2.54(1.04,6.19) |  | 2.36(0.95,5.85) |  |
| FO day 30 | 0-1 | 121 | 0.421(0.332,0.508) | Reference | | 0.78 | Reference | 0.87 | 0.116(0.066,0.180) | | Reference | 0.16 | Reference | 0.20 |
|  | 2 | 132 | 0.439(0.353,0.522) | 1.07(0.74,1.55) | |  | 1.07(0.74,1.55) |  | 0.144(0.090,0.210) | | 1.25(0.63,2.49) |  | 1.25(0.63,2.48) |  |
|  | 3-4 | 22 | 0.500(0.270,0.693) | 1.21(0.64,2.27) | |  | 1.16(0.61,2.23) |  | 0.273(0.107,0.471) | | 2.51(0.98,6.42) |  | 2.39(0.92,6.19) |  |
| Weight Δ | ≤5% | 137 | 0.431(0.346,0.512) | Reference | | 0.58 | Reference | 0.52 | 0.102(0.059,0.160) | | Reference | 0.17 | Reference | 0.21 |
|  | >5-10% | 96 | 0.469(0.366,0.565) | 1.13(0.77,1.65) | |  | 1.12(0.76,1.64) |  | 0.188(0.116,0.272) | | 1.91(0.95,3.84) |  | 1.87(0.93,3.76) |  |
|  | >10% | 42 | 0.381(0.235,0.526) | 0.85(0.51,1.44) | |  | 0.81(0.48,1.37) |  | 0.167(0.072,0.295) | | 1.66(0.68,4.06) |  | 1.55(0.63,3.77) |  |
| Weight Δ | Per 5% |  |  | 0.94(0.81,1.09) | | 0.42 | 0.93(0.79,1.08) | 0.33 |  | | 1.10(0.91,1.34) | 0.33 | 1.09(0.89,1.32) | 0.42 |

* Based on univariate analysis and Gray’s test

† Based on the multivariable Fine and Gray models adjusted for graft source.

|  | | | ***Any cGVHD*** | | | | | ***Extensive cGVHD*** | | | | |
| --- | --- | --- | --- | --- | --- | --- | --- | --- | --- | --- | --- | --- |
| ***Variable*** |  | ***N*** | ***1-Yr (95%CI)**** | ***HR (95%CI)**** | ***Gray P**** | ***Adjusted HR (95%CI)* †** | ***FG test P*†** | ***1 Yr (95%CI)**** | ***HR (95%CI)**** | ***Gray P**** | ***Adjusted HR (95%CI)* †** | ***FG test P*†** |
| Age, years | ≤17 | 26 | 0.423(0.229,0.605) | Reference | 0.32 | Reference | 0.17 | 0.346(0.170,0.530) | Reference | 0.54 | Reference | 0.41 |
|  | 18-39 | 81 | 0.346(0.244,0.450) | 0.87(0.46,1.66) |  | 0.77(0.40,1.48) |  | 0.272(0.180,0.372) | 0.77(0.38,1.58) |  | 0.64(0.30,1.37) |  |
|  | 40-59 | 90 | 0.483(0.375,0.583) | 1.28(0.70,2.35) |  | 1.13(0.60,2.14) |  | 0.348(0.251,0.448) | 1.07(0.54,2.10) |  | 1.00(0.46,2.16) |  |
|  | ≥60 | 78 | 0.359(0.253,0.466) | 0.93(0.49,1.75) |  | 0.72(0.37,1.39) |  | 0.256(0.165,0.358) | 0.79(0.39,1.60) |  | 0.86(0.37,2.03) |  |
|  | Per 5 yr |  |  | 1.00(0.96,1.05) | 0.87 | 0.98(0.93,1.02) | 0.32 |  | 0.99(0.94,1.05) | 0.79 | 1.01(0.94,1.09) | 0.69 |
| Sex | M | 161 | 0.412(0.335,0.488) | Reference | 0.64 | Reference | 0.14 | 0.288(0.219,0.359) | Reference | 0.22 | Reference | 0.069 |
|  | F | 114 | 0.386(0.296,0.475) | 1.09(0.76,1.55) |  | 1.36(0.90,2.06) |  | 0.316(0.232,0.402) | 1.29(0.86,1.94) |  | 1.54(0.97,2.46) |  |
| KPS | ≥80 | 237 | 0.419(0.356,0.482) | Reference | 0.42 | Reference | 0.33 | 0.318(0.259,0.378) | Reference | 0.50 | Reference | 0.43 |
|  | ≤70 | 38 | 0.289(0.154,0.440) | 0.80(0.47,1.37) |  | 0.78(0.47,1.29) |  | 0.184(0.080,0.323) | 0.80(0.43,1.50) |  | 0.78(0.42,1.44) |  |
| HCTCI | 0 | 74 | 0.446(0.330,0.556) | Reference | 0.63 | Reference | 0.32 | 0.324(0.220,0.432) | Reference | 0.81 | Reference | 0.44 |
|  | 1-2 | 89 | 0.375(0.274,0.476) | 0.83(0.54,1.29) |  | 0.74(0.48,1.14) |  | 0.250(0.165,0.345) | 0.84(0.50,1.42) |  | 0.71(0.42,1.21) |  |
|  | ≥3 | 112 | 0.393(0.302,0.482) | 0.82(0.53,1.27) |  | 0.76(0.49,1.17) |  | 0.321(0.237,0.409) | 0.94(0.57,1.54) |  | 0.86(0.52,1.41) |  |
|  | Per 1 |  |  | 0.97(0.87,1.07) | 0.53 | 0.97(0.87,1.08) | 0.57 |  | 0.99(0.88,1.11) | 0.88 | 0.99(0.87,1.12) | 0.87 |
| DRI | Low | 48 | 0.354(0.221,0.490) | Reference | 0.99 | Reference | 0.86 | 0.229(0.122,0.357) | Reference | 0.63 | Reference | 0.61 |
|  | Int-high | 202 | 0.413(0.344,0.480) | 1.00(0.63,1.59) |  | 0.98(0.62,1.55) |  | 0.313(0.250,0.378) | 1.25(0.71,2.20) |  | 1.26(0.72,2.18) |  |
|  | Non-malig | 25 | 0.400(0.208,0.586) | 1.04(0.53,2.06) |  | 1.16(0.58,2.30) |  | 0.320(0.149,0.506) | 1.45(0.66,3.18) |  | 1.48(0.65,3.36) |  |
| Conditioning | MAC | 121 | 0.458(0.367,0.545) | Reference | 0.24 | Reference | 0.13 | 0.375(0.288,0.461) | Reference | **0.042** | Reference | **0.037** |
|  | RIC/NMA | 154 | 0.357(0.282,0.433) | 0.81(0.57,1.15) |  | 0.76(0.54,1.08) |  | 0.240(0.176,0.310) | 0.66(0.44,0.99) |  | 0.65(0.43,0.97) |  |
| Graft | PBSC | 220 | 0.434(0.367,0.498) | Reference | 0.088 | Reference | 0.17 | 0.324(0.263,0.387) | Reference | 0.14 | Reference | 0.18 |
|  | BM | 55 | 0.273(0.162,0.395) | 0.66(0.40,1.09) |  | 0.70(0.42,1.17) |  | 0.200(0.106,0.315) | 0.66(0.37,1.17) |  | 0.67(0.38,1.21) |  |
| F to M | No | 224 | 0.363(0.300,0.426) | Reference | **0.005** | Reference | **0.007** | 0.274(0.217,0.333) | Reference | 0.11 | Reference | **0.019** |
|  | Yes | 51 | 0.569(0.419,0.693) | 1.76(1.19,2.60) |  | 1.75(1.17,2.61) |  | 0.412(0.275,0.544) | 1.48(0.92,2.39) |  | 1.94(1.12,3.37) |  |
| Donor age | ≤34 | 157 | 0.376(0.300,0.451) | Reference | 0.077 | Reference | **0.040** | 0.261(0.195,0.332) | Reference | **0.018** | Reference | **0.013** |
|  | ≥35 | 118 | 0.436(0.344,0.524) | 1.37(0.97,1.94) |  | 1.44(1.02,2.04) |  | 0.350(0.265,0.437) | 1.62(1.08,2.44) |  | 1.67(1.11,2.49) |  |
|  | Per 5 yr |  |  | 1.03(0.96,1.11) | 0.36 | 0.93(0.80,1.07) | 0.28 |  | 1.05(0.97,1.14) | 0.21 | 0.89(0.76,1.03) | 0.13 |
| Donor Type | Haplo | 210 | 0.397(0.330,0.463) | Reference | **0.024** | Reference | 0.073 | 0.297(0.236,0.360) | Reference | 0.31 | Reference | 0.29 |
|  | MRD/MUD | 21 | 0.190(0.057,0.383) | 0.46(0.19,1.15) |  | 0.44(0.18,1.10) |  | 0.190(0.057,0.383) | 0.68(0.27,1.70) |  | 0.62(0.25,1.52) |  |
|  | MMUD | 44 | 0.523(0.364,0.660) | 1.50(0.95,2.36) |  | 1.39(0.86,2.23) |  | 0.364(0.223,0.505) | 1.36(0.80,2.30) |  | 1.36(0.78,2.37) |  |
| HCT era | 2009-17 | 176 | 0.398(0.325,0.469) | Reference | 0.96 | Reference | 0.47 | 0.301(0.235,0.370) | Reference | 0.79 | Reference | 0.34 |
|  | 2018 | 99 | 0.408(0.310,0.504) | 0.98(0.68,1.40) |  | 0.87(0.60,1.26) |  | 0.296(0.209,0.388) | 0.93(0.61,1.42) |  | 0.81(0.53,1.24) |  |
| FO day 3-8 | 0-1 | 173 | 0.459(0.383,0.532) | Reference | **0.019** | Reference | **0.008** | 0.337(0.267,0.408) | Reference | 0.11 | Reference | 0.091 |
|  | 2 | 80 | 0.363(0.258,0.468) | 0.82(0.56,1.22) |  | 0.76(0.51,1.13) |  | 0.275(0.182,0.376) | 0.84(0.53,1.32) |  | 0.81(0.51,1.28) |  |
|  | 3-4 | 22 | 0.091(0.013,0.264) | 0.20(0.07,0.62) |  | 0.20(0.07,0.60) |  | 0.091(0.013,0.264) | 0.31(0.10,0.93) |  | 0.32(0.11,0.94) |  |
| FO day 30 | 0-1 | 121 | 0.467(0.375,0.553) | Reference | **0.027** | Reference | **0.020** | 0.308(0.228,0.392) | Reference | 0.12 | Reference | 0.098 |
|  | 2 | 132 | 0.394(0.310,0.477) | 0.90(0.63,1.29) |  | 0.89(0.63,1.27) |  | 0.326(0.247,0.407) | 1.15(0.76,1.74) |  | 1.21(0.80,1.85) |  |
|  | 3-4 | 22 | 0.091(0.013,0.264) | 0.21(0.07,0.63) |  | 0.20(0.07,0.62) |  | 0.091(0.013,0.264) | 0.35(0.11,1.08) |  | 0.39(0.13,1.14) |  |
| Weight Δ | ≤5% | 137 | 0.431(0.346,0.512) | Reference | 0.65 | Reference | 0.69 | 0.314(0.238,0.393) | Reference | 0.97 | Reference | 0.96 |
|  | >5-10% | 96 | 0.411(0.310,0.508) | 1.02(0.69,1.50) |  | 0.98(0.66,1.44) |  | 0.295(0.206,0.389) | 0.94(0.60,1.48) |  | 0.98(0.62,1.54) |  |
|  | >10% | 42 | 0.286(0.157,0.429) | 0.77(0.45,1.32) |  | 0.78(0.45,1.37) |  | 0.262(0.139,0.403) | 0.97(0.53,1.75) |  | 1.07(0.56,2.05) |  |
| Weight Δ | Per 5% |  |  | 0.85(0.72,1.00) | 0.057 | 0.82(0.67,0.99) | **0.039** |  | 0.91(0.76,1.09) | 0.30 | 0.94(0.77,1.14) | 0.52 |

* Based on univariate analysis and Gray’s test

† Based on the multivariable Fine and Gray models. Models on any cGVHD were adjusted for F to M HCT, donor type, donor age, and graft source. Models for extensive cGVHD were adjusted for recipient sex, conditioning and F to M HCT.
