# Supplementary material for: Impact of hyperhydration on fluid overload and hematopoietic cell transplant after post-transplant cyclophosphamide-based graft-versus-host-disease prophylaxis
Source: Front Immunol. 2025 Feb 20;16:1543099. doi: 10.3389/fimmu.2025.1543099 (PMC11882550; doi:10.3389/fimmu.2025.1543099)
Supplement: Supplementary file 2 [file Table2.docx]

Supplementary Table 2: Multivariate Analysis for NRM and Relapse

|  | | | ***Relapse*** | | | | | ***NRM*** | | | | | |
| --- | --- | --- | --- | --- | --- | --- | --- | --- | --- | --- | --- | --- | --- |
| ***Variable*** |  | ***N*** | ***2 Yr (95%CI)**** | ***HR (95%CI)**** | ***Gray P**** | ***Adjusted HR (95%CI)†*** | ***FG test P†*** | ***100 day (95%CI)**** | ***1 Yr (95%CI)**** | ***HR (95%CI)**** | ***Gray P**** | ***Adjusted HR (95%CI)†*** | ***FG test P†*** |
| Age, years | ≤17 | 26 | 0.115(0.028,0.271) | Reference | 0.46 | Reference | 0.50 | 0.0 (no events) | 0.038(0.003,0.168) | Reference | **<0.001** | Reference | **<0.001** |
|  | 18-39 | 81 | 0.247(0.159,0.345) | 2.56(0.74,8.85) |  | 2.41(0.62,9.34) |  | 0.025(0.005,0.078) | 0.086(0.038,0.160) | 3.78(0.49,29.19) |  | 3.92(0.50,30.66) |  |
|  | 40-59 | 90 | 0.189(0.115,0.276) | 2.27(0.66,7.85) |  | 1.92(0.48,7.61) |  | 0.067(0.027,0.131) | 0.178(0.107,0.264) | 7.45(1.01,55.08) |  | 8.49(1.12,64.48) |  |
|  | ≥60 | 78 | 0.205(0.123,0.302) | 2.11(0.60,7.39) |  | 1.72(0.43,6.91) |  | 0.154(0.084,0.243) | 0.346(0.242,0.452) | 14.01(1.92,102.37) |  | 14.36(1.94,106.41) |  |
|  | Per 5 yr |  |  | 1.01(0.95,1.07) | 0.83 | 0.98(0.91,1.05) | 0.59 |  |  | 1.20(1.12,1.29) | **<0.001** | 1.20(1.11,1.29) | **<0.001** |
| Sex | M | 161 | 0.230(0.168,0.298) | Reference | 0.19 | Reference | 0.14 | 0.056(0.027,0.099) | 0.193(0.136,0.257) | Reference | 0.51 | Reference | 0.24 |
|  | F | 114 | 0.167(0.105,0.241) | 0.72(0.44,1.19) |  | 0.68(0.41,1.13) |  | 0.096(0.051,0.159) | 0.175(0.112,0.251) | 1.17(0.73,1.90) |  | 1.35(0.82,2.21) |  |
| KPS | ≥80 | 237 | 0.198(0.150,0.251) | Reference | 0.61 | Reference | 0.28 | 0.072(0.043,0.109) | 0.165(0.120,0.215) | Reference | **0.016** | Reference | **0.020** |
|  | ≤70 | 38 | 0.237(0.115,0.383) | 1.18(0.60,2.34) |  | 1.46(0.73,2.94) |  | 0.079(0.020,0.193) | 0.316(0.175,0.467) | 2.03(1.15,3.57) |  | 2.01(1.11,3.63) |  |
| HCTCI | 0 | 74 | 0.135(0.069,0.224) | Reference | 0.50 | Reference | 0.50 | 0.054(0.017,0.122) | 0.122(0.059,0.208) | Reference | 0.087 | Reference | 0.21 |
|  | 1-2 | 89 | 0.191(0.117,0.279) | 1.04(0.54,2.00) |  | 1.29(0.66,2.52) |  | 0.067(0.027,0.132) | 0.213(0.135,0.304) | 2.07(1.02,4.23) |  | 1.82(0.88,3.75) |  |
|  | ≥3 | 112 | 0.259(0.182,0.343) | 1.37(0.76,2.47) |  | 1.42(0.79,2.57) |  | 0.089(0.046,0.151) | 0.205(0.136,0.285) | 1.96(0.97,3.97) |  | 1.83(0.90,3.71) |  |
|  | Per 1 |  |  | 1.08(0.96,1.21) | 0.20 | 1.07(0.96,1.19) | 0.21 |  |  | 1.08(0.96,1.20) | 0.19 | 1.04(0.93,1.17) | 0.45 |
| DRI | Low | 48 | 0.146(0.063,0.261) | Reference | **0.003** | Reference | **<0.001** | 0.0 (no events) | 0.125(0.050,0.236) | Reference | **0.015** | Reference | 0.20 |
|  | Int-high | 202 | 0.243(0.186,0.304) | 1.99(0.88,4.49) |  | 2.29(0.99,5.31) |  | 0.099(0.063,0.145) | 0.218(0.164,0.277) | 2.18(1.00,4.74) |  | 2.03(0.91,4.54) |  |
|  | Non-malig | 25 | 0.0 (no events) | 0.00(0.00,0.00) |  | 0.00(0.00,0.00) |  | 0.0 (no events) | 0.040(0.003,0.174) | 0.54(0.11,2.51) |  | 1.33(0.25,7.17) |  |
| Conditioning | MAC | 121 | 0.190(0.126,0.265) | Reference | 0.96 | Reference | 0.43 | 0.033(0.011,0.077) | 0.107(0.060,0.170) | Reference | **0.002** | Reference | 0.47 |
|  | RIC/NMA | 154 | 0.214(0.153,0.282) | 1.02(0.63,1.67) |  | 0.82(0.50,1.35) |  | 0.104(0.062,0.158) | 0.247(0.182,0.317) | 2.32(1.36,3.98) |  | 1.25(0.68,2.29) |  |
| Graft | PBSC | 220 | 0.182(0.134,0.236) | Reference | **0.047** | Reference | **<0.001** | 0.086(0.054,0.128) | 0.209(0.158,0.265) | Reference | 0.072 | Reference | 0.56 |
|  | BM | 55 | 0.291(0.177,0.415) | 1.76(1.03,3.01) |  | 2.73(1.60,4.65) |  | 0.018(0.001,0.086) | 0.091(0.033,0.185) | 0.55(0.28,1.08) |  | 0.81(0.41,1.62) |  |
| F to M | No | 224 | 0.196(0.147,0.251) | Reference | 0.53 | Reference | 0.46 | 0.080(0.049,0.121) | 0.183(0.135,0.236) | Reference | 0.84 | Reference | 0.78 |
|  | Yes | 51 | 0.235(0.129,0.360) | 1.21(0.68,2.16) |  | 1.24(0.69,2.24) |  | 0.039(0.007,0.120) | 0.196(0.100,0.316) | 1.06(0.58,1.92) |  | 0.92(0.50,1.70) |  |
| Donor age | ≤34 | 157 | 0.229(0.167,0.298) | Reference | 0.47 | Reference | 0.53 | 0.089(0.051,0.140) | 0.172(0.118,0.235) | Reference | 0.51 | Reference | 0.93 |
|  | ≥35 | 118 | 0.170(0.108,0.243) | 0.84(0.51,1.36) |  | 0.85(0.52,1.39) |  | 0.051(0.021,0.101) | 0.203(0.136,0.280) | 1.17(0.72,1.88) |  | 1.02(0.62,1.68) |  |
|  | Per 5 yr |  |  | 0.98(0.89,1.09) | 0.75 | 0.97(0.86,1.08) | 0.57 |  |  | 1.05(0.95,1.15) | 0.34 | 1.02(0.92,1.13) | 0.72 |
| Donor Type | Haplo | 210 | 0.229(0.174,0.288) | Reference | 0.16 | Reference | 0.20 | 0.081(0.049,0.123) | 0.190(0.140,0.246) | Reference | 0.48 | Reference | 0.70 |
|  | MRD/MUD | 21 | 0.143(0.034,0.326) | 0.53(0.16,1.72) |  | 0.52(0.15,1.79) |  | 0.0 (no events) | 0.095(0.015,0.267) | 0.55(0.18,1.70) |  | 0.63(0.21,1.88) |  |
|  | MMUD | 44 | 0.114(0.041,0.228) | 0.49(0.21,1.15) |  | 0.52(0.22,1.22) |  | 0.068(0.017,0.169) | 0.205(0.100,0.335) | 1.17(0.63,2.18) |  | 0.93(0.48,1.78) |  |
| HCT era | 2009-17 | 176 | 0.227(0.168,0.292) | Reference | 0.16 | Reference | 0.42 | 0.085(0.050,0.132) | 0.199(0.143,0.261) | Reference | 0.39 | Reference | 0.50 |
|  | 2018 | 99 | 0.162(0.097,0.241) | 0.67(0.39,1.15) |  | 0.79(0.46,1.38) |  | 0.051(0.019,0.107) | 0.162(0.097,0.241) | 0.78(0.46,1.31) |  | 0.83(0.48,1.44) |  |
| FO day 3-8 | 0-1 | 173 | 0.237(0.176,0.303) | Reference | 0.26 | Reference | 0.46 | 0.017(0.005,0.046) | 0.121(0.078,0.175) | Reference | **<0.001** | Reference | **<0.001** |
|  | 2 | 80 | 0.162(0.091,0.252) | 0.71(0.40,1.24) |  | 0.82(0.47,1.43) |  | 0.088(0.038,0.162) | 0.213(0.130,0.308) | 1.82(1.07,3.11) |  | 1.38(0.81,2.35) |  |
|  | 3-4 | 22 | 0.091(0.014,0.262) | 0.47(0.15,1.47) |  | 0.52(0.16,1.65) |  | 0.455(0.238,0.649) | 0.591(0.351,0.768) | 6.78(3.32,13.85) |  | 5.67(2.79,11.51) |  |
| FO day 30 | 0-1 | 121 | 0.248(0.175,0.328) | Reference | 0.38 | Reference | 0.52 | 0.017(0.003,0.053) | 0.099(0.054,0.160) | Reference | **<0.001** | Reference | **<0.001** |
|  | 2 | 132 | 0.182(0.121,0.252) | 0.81(0.49,1.33) |  | 0.88(0.54,1.43) |  | 0.061(0.028,0.110) | 0.197(0.134,0.269) | 1.94(1.11,3.38) |  | 1.55(0.88,2.71) |  |
|  | 3-4 | 22 | 0.091(0.014,0.262) | 0.46(0.15,1.48) |  | 0.51(0.16,1.67) |  | 0.455(0.238,0.649) | 0.591(0.351,0.768) | 8.01(3.71,17.29) |  | 6.51(3.02,14.03) |  |
| Weight Δ | ≤5% | 137 | 0.226(0.160,0.300) | Reference | 0.14 | Reference | 0.16 | 0.007(0.001,0.037) | 0.080(0.042,0.134) | Reference | **<0.001** | Reference | **<0.001** |
|  | >5-10% | 96 | 0.229(0.151,0.318) | 1.19(0.72,1.97) |  | 1.20(0.72,1.98) |  | 0.063(0.025,0.123) | 0.167(0.100,0.248) | 1.66(0.91,3.01) |  | 1.43(0.79,2.57) |  |
|  | >10% | 42 | 0.071(0.018,0.178) | 0.45(0.18,1.13) |  | 0.48(0.19,1.21) |  | 0.310(0.177,0.452) | 0.571(0.406,0.707) | 6.86(3.79,12.41) |  | 4.48(2.39,8.41) |  |
| Weight Δ | Per 5% |  |  | 0.87(0.70,1.08) | 0.20 | 0.87(0.71,1.07) |  |  |  | 2.06(1.76,2.41) | **<0.001** | 1.91(1.64,2.23) | **<0.001** |

* Based on univariate analysis and Gray’s test

† Based on the multivariable Fine and Gray models. Models on relapse were adjusted for DRI and graft type. Models on NRM were adjusted for recipient age per 5 years and KPS.
