# Supplementary material for: Impact of hyperhydration on fluid overload and hematopoietic cell transplant after post-transplant cyclophosphamide-based graft-versus-host-disease prophylaxis
Source: Front Immunol. 2025 Feb 20;16:1543099. doi: 10.3389/fimmu.2025.1543099 (PMC11882550; doi:10.3389/fimmu.2025.1543099)
Supplement: Supplementary file 3 [file Table3.docx]

Supplementary Material

Supplementary Table 3: Multivariate Analysis for OS and DFS

|  | | | ***Overall Survival*** | | | | | ***DFS*** | | | | |
| --- | --- | --- | --- | --- | --- | --- | --- | --- | --- | --- | --- | --- |
| ***Variable*** |  | ***N*** | ***2 Yr (95%CI)**** | ***HR (95%CI)**** | ***P**** | ***Adjusted HR (95%CI)†*** | ***Wald test P†*** | ***2 Yr (95%CI)**** | ***HR (95%CI)**** | ***P**** | ***Adjusted HR (95%CI)†*** | ***Wald test P†*** |
| Age, years | ≤17 | 26 | 0.885(0.684,0.961) | Reference | **<0.001** | Reference | **0.011** | 0.846(0.640,0.939) | Reference | **<0.001** | Reference | **0.020** |
|  | 18-39 | 81 | 0.728(0.617,0.812) | 3.40(1.04,11.16) |  | 2.25(0.66,7.63) |  | 0.654(0.540,0.747) | 3.05(1.08,8.60) |  | 2.32(0.79,6.78) |  |
|  | 40-59 | 90 | 0.622(0.514,0.713) | 4.72(1.46,15.22) |  | 2.79(0.82,9.47) |  | 0.578(0.469,0.672) | 3.84(1.38,10.68) |  | 2.68(0.91,7.90) |  |
|  | ≥60 | 78 | 0.474(0.361,0.580) | 7.84(2.45,25.14) |  | 4.27(1.27,14.42) |  | 0.410(0.301,0.516) | 6.11(2.21,16.90) |  | 3.89(1.33,11.39) |  |
|  | Per 5 yr |  |  | 1.13(1.07,1.19) | **<0.001** | 1.09(1.03,1.16) | **0.003** |  | 1.11(1.06,1.17) | **<0.001** | 1.07(1.02,1.13) | **0.009** |
| Sex | M | 161 | 0.627(0.548,0.697) | Reference | 0.90 | Reference | 0.49 | 0.559(0.479,0.632) | Reference | 0.54 | Reference | 0.58 |
|  | F | 114 | 0.649(0.554,0.729) | 1.02(0.71,1.48) |  | 1.14(0.79,1.65) |  | 0.605(0.509,0.688) | 0.90(0.63,1.27) |  | 0.91(0.64,1.29) |  |
| KPS | ≥80 | 237 | 0.662(0.598,0.719) | Reference | **0.003** | Reference | **0.002** | 0.603(0.538,0.662) | Reference | **0.006** | Reference | **<0.001** |
|  | ≤70 | 38 | 0.474(0.310,0.621) | 1.95(1.24,3.06) |  | 2.08(1.32,3.29) |  | 0.421(0.264,0.570) | 1.82(1.17,2.81) |  | 2.33(1.49,3.65) |  |
| HCTCI | 0 | 74 | 0.770(0.657,0.850) | Reference | **0.017** | Reference | 0.12 | 0.729(0.613,0.816) | Reference | **0.029** | Reference | **0.049** |
|  | 1-2 | 89 | 0.652(0.543,0.741) | 1.62(0.97,2.72) |  | 1.38(0.82,2.32) |  | 0.573(0.464,0.668) | 1.61(0.99,2.61) |  | 1.66(1.01,2.72) |  |
|  | ≥3 | 112 | 0.536(0.439,0.623) | 2.00(1.23,3.26) |  | 1.67(1.02,2.72) |  | 0.482(0.387,0.571) | 1.84(1.16,2.93) |  | 1.77(1.11,2.83) |  |
|  | Per 1 |  |  | 1.12(1.02,1.22) | **0.012** | 1.07(0.98,1.17) | 0.11 |  | 1.10(1.01,1.19) | **0.029** | 1.08(0.99,1.18) | 0.072 |
| DRI | Low | 48 | 0.771(0.625,0.866) | Reference | **<0.001** | Reference | **0.002** | 0.708(0.558,0.816) | Reference | **<0.001** | Reference | **<0.001** |
|  | Int-high | 202 | 0.569(0.498,0.634) | 2.44(1.34,4.43) |  | 2.24(1.23,4.08) |  | 0.505(0.434,0.571) | 2.30(1.32,4.00) |  | 2.26(1.29,3.95) |  |
|  | Non-malig | 25 | 0.920(0.716,0.979) | 0.29(0.07,1.30) |  | 0.38(0.08,1.82) |  | 0.920(0.716,0.979) | 0.23(0.05,1.02) |  | 0.14(0.03,0.65) |  |
| Conditioning | MAC | 121 | 0.727(0.638,0.798) | Reference | **<0.001** | Reference | 0.073 | 0.686(0.595,0.761) | Reference | **0.005** | Reference | **0.023** |
|  | RIC/NMA | 154 | 0.565(0.483,0.639) | 1.90(1.29,2.81) |  | 1.51(0.96,2.38) |  | 0.494(0.412,0.569) | 1.66(1.16,2.39) |  | 1.53(1.06,2.22) |  |
| Graft | PBSC | 220 | 0.632(0.564,0.692) | Reference | 0.56 | Reference | 0.12 | 0.582(0.514,0.644) | Reference | 0.98 | Reference | **0.045** |
|  | BM | 55 | 0.655(0.513,0.764) | 0.88(0.56,1.37) |  | 1.45(0.91,2.33) |  | 0.563(0.422,0.682) | 0.99(0.65,1.52) |  | 1.56(1.01,2.42) |  |
| F to M | No | 224 | 0.638(0.572,0.697) | Reference | 0.85 | Reference | 0.75 | 0.589(0.522,0.650) | Reference | 0.53 | Reference | 0.67 |
|  | Yes | 51 | 0.627(0.480,0.744) | 1.05(0.66,1.65) |  | 0.93(0.59,1.47) |  | 0.529(0.385,0.655) | 1.14(0.75,1.75) |  | 1.10(0.72,1.68) |  |
| Donor age | ≤34 | 157 | 0.656(0.576,0.725) | Reference | 0.93 | Reference | 0.83 | 0.580(0.498,0.652) | Reference | 0.94 | Reference | 0.88 |
|  | ≥35 | 118 | 0.610(0.516,0.691) | 1.02(0.71,1.46) |  | 1.04(0.72,1.51) |  | 0.576(0.482,0.659) | 0.99(0.70,1.39) |  | 1.03(0.73,1.46) |  |
|  | Per 5 yr |  |  | 1.02(0.94,1.10) | 0.64 | 1.02(0.94,1.10) | 0.63 |  | 1.02(0.95,1.09) | 0.63 | 1.02(0.95,1.10) | 0.59 |
| Donor Type | Haplo | 210 | 0.624(0.554,0.685) | Reference | 0.30 | Reference | 0.31 | 0.557(0.487,0.621) | Reference | 0.18 | Reference | 0.27 |
|  | MRD/MUD | 21 | 0.762(0.519,0.893) | 0.50(0.20,1.23) |  | 0.60(0.24,1.49) |  | 0.714(0.472,0.860) | 0.50(0.22,1.13) |  | 0.58(0.25,1.31) |  |
|  | MMUD | 44 | 0.636(0.477,0.759) | 0.92(0.55,1.55) |  | 0.74(0.44,1.24) |  | 0.614(0.454,0.739) | 0.80(0.48,1.31) |  | 0.76(0.45,1.27) |  |
| HCT era | 2009-17 | 176 | 0.597(0.520,0.665) | Reference | 0.11 | Reference | 0.18 | 0.540(0.463,0.610) | Reference | **0.049** | Reference | 0.22 |
|  | 2018 | 99 | 0.707(0.607,0.786) | 0.72(0.48,1.09) |  | 0.75(0.49,1.14) |  | 0.646(0.544,0.732) | 0.69(0.47,1.01) |  | 0.78(0.52,1.16) |  |
| FO day 3-8 | 0-1 | 173 | 0.682(0.607,0.746) | Reference | **<0.001** | Reference | **<0.001** | 0.618(0.542,0.686) | Reference | **<0.001** | Reference | **<0.001** |
|  | 2 | 80 | 0.625(0.509,0.721) | 1.29(0.86,1.94) |  | 1.13(0.74,1.72) |  | 0.575(0.459,0.675) | 1.19(0.81,1.75) |  | 1.22(0.82,1.80) |  |
|  | 3-4 | 22 | 0.318(0.142,0.511) | 3.55(2.02,6.25) |  | 3.48(1.94,6.24) |  | 0.273(0.111,0.464) | 3.35(1.97,5.68) |  | 4.05(2.37,6.92) |  |
| FO day 30 | 0-1 | 121 | 0.711(0.621,0.783) | Reference | **<0.001** | Reference | **<0.001** | 0.636(0.544,0.715) | Reference | **<0.001** | Reference | **<0.001** |
|  | 2 | 132 | 0.621(0.533,0.698) | 1.30(0.88,1.92) |  | 1.19(0.80,1.78) |  | 0.576(0.487,0.655) | 1.24(0.86,1.80) |  | 1.24(0.85,1.81) |  |
|  | 3-4 | 22 | 0.318(0.142,0.511) | 3.77(2.09,6.79) |  | 3.68(2.00,6.77) |  | 0.273(0.111,0.464) | 3.56(2.05,6.18) |  | 4.28(2.44,7.51) |  |
| Weight Δ | ≤5% | 137 | 0.723(0.639,0.790) | Reference | **<0.001** | Reference | **<0.001** | 0.657(0.571,0.730) | Reference | **<0.001** | Reference | **<0.001** |
|  | >5-10% | 96 | 0.635(0.531,0.723) | 1.29(0.85,1.96) |  | 1.11(0.72,1.70) |  | 0.573(0.468,0.665) | 1.49(1.01,2.21) |  | 1.39(0.93,2.06) |  |
|  | >10% | 42 | 0.357(0.217,0.499) | 3.70(2.33,5.88) |  | 2.84(1.72,4.67) |  | 0.333(0.198,0.475) | 3.10(1.97,4.88) |  | 3.04(1.90,4.89) |  |
| Weight Δ | Per 5% |  |  | 1.89(1.64,2.19) | **<0.001** | 1.75(1.49,2.04) | **<0.001** |  | 1.77(1.53,2.04) | **<0.001** | 1.70(1.46,1.97) | **<0.001** |

* Based on univariate analysis and log-rank test

† Based on the multivariable Cox proportional hazards models. Models on OS were adjusted for recipient age per 5 years, KPS and DRI. Models on DFS were adjusted for KPS, DRI and graft source.
